# A Common Carcinogen Benzo[a]pyrene Causes Neuronal Death in Mouse via Microglial Activation

**DOI:** 10.1371/journal.pone.0009984

**Published:** 2010-04-01

**Authors:** Kallol Dutta, Debapriya Ghosh, Arshed Nazmi, Kanhaiya Lal Kumawat, Anirban Basu

**Affiliations:** National Brain Research Centre, Manesar, Haryana, India; Julius-Maximilians-Universität Würzburg, Germany

## Abstract

**Background:**

Benzo[a]pyrene (B[a]P) belongs to a class of polycyclic aromatic hydrocarbons that serve as micropollutants in the environment. B[a]P has been reported as a probable carcinogen in humans. Exposure to B[a]P can take place by ingestion of contaminated (especially grilled, roasted or smoked) food or water, or inhalation of polluted air. There are reports available that also suggests neurotoxicity as a result of B[a]P exposure, but the exact mechanism of action is unknown.

**Methodology/Principal Findings:**

Using neuroblastoma cell line and primary cortical neuron culture, we demonstrated that B[a]P has no direct neurotoxic effect. We utilized both *in vivo* and *in vitro* systems to demonstrate that B[a]P causes microglial activation. Using microglial cell line and primary microglial culture, we showed for the first time that B[a]P administration results in elevation of reactive oxygen species within the microglia thereby causing depression of antioxidant protein levels; enhanced expression of inducible nitric oxide synthase, that results in increased production of NO from the cells. Synthesis and secretion of proinflammatory cytokines were also elevated within the microglia, possibly via the p38MAP kinase pathway. All these factors contributed to bystander death of neurons, *in vitro*. When administered to animals, B[a]P was found to cause microglial activation and astrogliosis in the brain with subsequent increase in proinflammatory cytokine levels.

**Conclusions/Significance:**

Contrary to earlier published reports we found that B[a]P has no direct neurotoxic activity. However, it kills neurons in a bystander mechanism by activating the immune cells of the brain *viz* the microglia. For the first time, we have provided conclusive evidence regarding the mechanism by which the micropollutant B[a]P may actually cause damage to the central nervous system. In today's perspective, where rising pollution levels globally are a matter of grave concern, our study throws light on other health hazards that such pollutants may exert.

## Introduction

Benzo[a]pyrene (B[a]P), is a member of the polycyclic aromatic hydrocarbon (PAH) family that contains about 100 different chemicals; B[a]P being the most studied member of that family [1]. Owing to its relatively high environmental levels and high level of toxicity that results in larger health impact than any other PAH identified in the environment, B[a]P is often considered as a surrogate for other PAH compounds.

B[a]P is released into the environment (air, water and soil) from natural sources such as volcanoes, forest fires and from man-made sources including industrial and automobile exhaust fumes [2], manufacturing of products such as coal, tar, asphalt [3], petroleum, cigarette smoke [4], [5], [6] and charcoal-broiled, fried, roasted and smoked foods [7], [8], [9]. PAH levels in soil have been found to be higher in urban setup when compared to rural environment, due to vehicular traffic [10]. The general population is exposed to B[a]P on a daily basis (*Table 1*), mainly via ingestion of contaminated foods, water and inhalation of polluted air [11], [12], [13]. Lioy et. al. reported in 1988 that the estimated B[a]P intake ranges from 20 to 800 ng per day in people living in the vicinity of hazardous waste sites contaminated by PAHs [14]. In 1991, Hattemer-Frey and Travis reported that the long-term average daily intake of B[a]P by the general population of the United States is estimated to be 2.2 µg per day [15]. The food ingestion pathway of accumulating PAHs such as B[a]P has been shown to be up to ten times higher in magnitude than risks determined from exposures due to other pathways such as soil contamination [16]. Cooked meat products have been shown to contain up to 4 ng/g of B[a]P [17], and up to 5.5 ng/g in fried chicken [18] and 62.6 ng/g in overcooked charcoal barbecued beef [19]. Fats and oils are also considered as one of the major sources of PAHs like B[a]P in the diet because of their lipophilic nature [20], [21], [22].

**Table 1:** Daily exposure range of humans to benzo[a]pyrene.

Source	Exposure amounts
Ingestion of unprocessed grain; charcoal-cooked or smoked meat	0.001–0.9 µg/day ^[9], [14]^
Ambient air exposure	0.02–3 µg/day ^[5], [12], [13]^
Ingestion from contaminated drinking water	0.002–0.12 mg/day ^[9]^
Inhalation of tobacco smoke (depending upon number of cigarettes smoked)	0.5–7.8 µg/day ^[5]^
Occupational exposure levels (via skin)	19.4–25.0 mg/day ^[3], [12], [14]^

The role of B[a]P in carcinogenesis is well established in animal models, but in humans, there are contradictions. The International Agency for Research on Cancer (IARC) and the Environmental Protection Agency (EPA) has determined that B[a]P is probably carcinogenic to humans [9]. B[a]P is thought to exert its carcinogenic effect via enzymatic activation into reactive metabolites that are capable of binding to the DNA, leading to uncontrolled proliferation [23], [24]. Apart from it's carcinogenic effects, B[a]P has also been shown to be involved in the development and progression of cardiovascular diseases [24], [25]. Owing to its lipophilic nature, B[a]P readily crosses blood-brain barrier and has also been shown to be accumulated and metabolized in the brain [26], [27] and there have been some studies that show neurotoxic effects *in vivo* [28], [29], [30], [31]. Due to high lipid content, rapid metabolic rate and decreased levels of antioxidant enzymes in the nervous system, it has been hypothesized that the nervous system may be highly susceptible to damage by B[a]P [32], though the exact mechanism that leads to damage to neurons in the central nervous system (CNS) is yet to be elucidated. From a behavioral aspect, B[a]P has been shown to affect neuromuscular, autonomic, sensorimotor and physiological functions [32] and also affects CNS development and plasticity [33]. Apart from direct damage, neurons are also susceptible to inflammatory damages resulting from glial cell activation in the brain. However, there is little published information relating B[a]P with modulation of glial cell activation. A study by Weng et al in 2004 had reported elevation of cyclooxygenase-2 in astrocytes following treatment by B[a]P metabolite to be mediated via nuclear factor kappa B (NFκB) [34], which suggests modulation of inflammatory response within the cell. Apart from that, till date there has been no scientific investigation in this area.

From a global perspective, with ever increasing environmental pollution levels, PAH contamination poses a serious concern. Since B[a]P is a potent toxic PAH that causes health hazards, the present investigation was undertaken to elucidate the underlying mechanism(s) by which B[a]P exerts its neurotoxic effect. We hypothesize that the neurotoxic effect of B[a]P, as observed in earlier studies, is inflammatory in nature, that is mediated by the activation of glial cells. Microglia being one of the major inflammatory cells of the CNS, we investigated the changes undergoing in them, on exposure to B[a]P, and the resulting changes in neuron-microglia interaction.

## Materials and Methods

### Ethics Statement

All animal experiments were approved by the institutional animal ethical review board named “Institutional Animal and Ethics Committee of National Brain Research Centre”. The animal experiment protocol approval no. is NBRC/IAEC/2008/46. Animals were handled in strict accordance with good animal practice as defined by Committee for the Purpose of Control and Supervision of Experiments on Animals (CPCSEA), Ministry of Environment and Forestry, Government of India.

### Cell lines and animals

Mouse neuroblastoma Neuro2a (N2a) cells were obtained from National Centre for Cell Science, Pune, India. Mouse microglial cell line BV-2 was a kind gift from Dr. Steve Levison, University of Medicine and Dentistry, New Jersey, USA. All the cell lines were grown at 37°C in DMEM supplemented with 3.7% sodium bi-carbonate (NaHCO_3_), 10% fetal bovine serum and penicillin/streptomycin. All the reagents related to cell culture were obtained from Sigma, St. Louis, USA, unless otherwise stated. 4–5 weeks old BALB/c mice of either sex, chosen randomly from a colony, were used for all *in vivo* experiments.

### Primary cell culture

Cortical neurons were cultured following a published protocol [35]. Briefly, cortices of P2 BALB/c mouse pups were dissected aseptically in calcium-magnesium-free (CMF)-Tyrode solution following decapitation. The meninges were removed, tissue were chopped into smaller pieces and collected in CMF-Tyrode. These were treated with trypsin DNAse and then dissociated in the same solution by triturating to make a single cell suspension, pelleted and resuspended in Neurobasal media. Neurobasal media was supplemented with L-glutamine (2 mM), 30% glucose, 5% fetal calf serum, 10% horse serum and penicillin–streptomycin. Cells were plated at a density of 5×10^3^ cells/cm^2^ onto poly-D-lysine-coated Labtek chamber slides and 96-well plate (Nunc, Roskilde, Denmark). After 48 h of incubation at 37°C, the serum containing medium was removed. Cells were incubated with serum free media for 4 h with antibiotics alone. For experimental treatments, the resting medium was exchanged for DMEM with N2 and B27 supplements, 25 mM KCl and antibiotics. Arabinoside (2×10^−5^ M) was used for the inhibition of astrocyte multiplication.

Primary mixed glial cultures were prepared from P0-2 mouse brains as described elsewhere [36], [37]. Briefly, BALB/c mouse pups were sacrificed by decapitation and whole brains excluding cerebelli and olfactory bulbs were isolated. The meninges were removed, tissues were enzymatically digested using trypsin-DNAse, mechanically dissociated, and the cell suspension was passed through 100 µm cell strainers before centrifuging at 400 g for 7.5 min. After triplicate counts with hemocytometer, cells were plated into 75-cm^2^ tissue culture flasks at a density of 2×10^5^ viable cells/cm^2^ in minimum essential medium (MEM; Invitrogen) supplemented with 10% fetal bovine serum, 2 mM glutamine, 100 U/100 lg/mL penicillin/streptomycin, and 0.6% glucose. Media was changed every 3 days after plating. On day 12, the mixed glial cultures were shaken on an Excella E25 (New Brunswick Scientific, NJ, USA) orbital shaker at 250 rpm for 60–75 min to dislodge microglial cells. The non-adherent cells after shaking were plated onto 6-well plates or chamber slides at 8×10^4^ viable cells/cm^2^, and incubated at 37°C for 30 min to allow microglial cells to adhere. The wells were rinsed extensively with serum free MEM to eliminate non-adherent cells and debris. The enriched microglial cultures were fed with 2 mL/well (for 6-well plates) or 1 mL/well (for 12-well plates) of hormone-supplemented media that contained 1% fetal bovine serum, 0.66 mg/mL bovine serum albumin (BSA), 100 µg/mL D-biotin, 5 ng/mL insulin, 1 ng/mL selenium, 40 lg/mL iron poor transferrin, 2 mM glutamine, 15 mM HEPES buffer, and 100 U/100 µg/mL penicillin/streptomycin.

### Determination of cell viability

Cell viability was assessed using the [3-(4,5-dimethylthiazol-2-yl)-5-(3-carboxymethoxyphenyl)-2-(4-sulfophenyl)-2H-tetrazolium, inner salt] (MTS; Promega, USA) assay as described earlier [38]. Mouse N2a and BV-2 cells were plated onto separate 96-well plates at a density of 2×10^4^ cells/well in triplicate. Cells were treated with varying doses of B[a]P (Sigma Aldrich, St. Louis, USA; 2.5, 5, 10, 20, 30, 40 and 80 µM) for 48 h. Cells marked as control were treated with DMSO. Twenty microliters of MTS solution was then added in each well. After 4 h incubation, the absorbance, reflecting the reduction of MTS by viable cells was determined at 490 nm. Values were expressed as a percentage relative to those obtained in controls.

To determine the effect of B[a]P on viability of primary neurons, cells were isolated as described above and then plated onto 96-well plates at a density of 2×10^4^ cells/well in triplicate. After the primary neurons developed processes, they were treated with B[a]P with similar doses, as done with N2a cells. MTS assay was then performed as described above.

To assess bystander neuronal death by factors released by microglial cells following B[a]P treatment, BV-2 cells were seeded in 60 mm culture plates at an density of 5×10^5^ cells/plate. After the cells became confluent, they were incubated with serum free DMEM for 6 h and then treated with three different doses of B[a]P (0.02, 0.2 and 2.0 µM) for 3, 12, 24 and 48 h. The supernatants were collected, filtered and added onto primary neurons cultured in poly-D-lysine coated 96 well plates. These cells were then incubated for 48 h following which 20 µL of MTS solution was added to each well and processed as described above.

### Measurement of ROS

The level of ROS produced within cells of control and each treatment groups were measured by the cell permeable, non-polar, H_2_O_2_-sensitive probe 5(and 6)-chlromethyl-20,70-dichlorodihydrofluoresceindiacetate (CM-H2DCFDA; Sigma, USA) by the method described previously [38]. CM-H2DCFDA diffuses into cells, where its acetate groups are cleaved by intracellular esterases, releasing the corresponding dichlorodihydrofluorescein derivative. Subsequent oxidations of CM-H2DCFDA yields a fluorescent adduct, dichlorofluorescein that is trapped inside the cell [39]. Briefly, BV-2 cells were plated in five 90-mm plates at a density of 5×10^5^ cells/mL (after triplicate counts with a haemocytometer) and were treated with three different doses of B[a]P (0.02, 0.2 and 2.0 µM) and incubated for 12 and 24 h. The cells were then harvested by washing with ice-cold PBS and then treated with 5 µM solution of CM-H2DCFDA followed by incubation in dark at room temperature for 45 min. Cells were then lysed with a buffer (lysis buffer) containing 1% Triton-X-100, 10 mM Tris–HCl (pH 8.0), 150 mM NaCl, 0.5% Nonidet P (NP-40), 1 mM EDTA, 0.2% EGTA, 0.2% sodium orthovanadate, and protease inhibitor cocktail (Sigma, USA) followed by centrifugation at 13000 rpm for 20 min at 4°C. The protein isolated was then used to measure the relative fluorescence with the help of Varioskan Flash multimode reader (Thermo Electron, Finland) at excitation 500 nm and emission 530 nm. The fluorescence intensity of intracellular CM-H2DCFDA is a linear indicator of the amount of H_2_O_2_ in the cells. A separate cell aliquote, without the CM-H2DCFDA, was lysed separately to estimate the protein content by Bradford method. The measured fluorescence intensity of was normalized to equal concentrations of protein in each sample.

### Measurement of Nitiric oxide released

Nitric oxide released from the microglial cells following B[a]P (0.02, 0.2 and 2.0 µM) treatment was assed using Griess reagent as described previously [35]. Briefly, BV-2 cells were cultured in 96-well plates at a density of 2×10^4^ cells/ml (after triplicate counts with a haemocytometer) and were treated as described above. Following 12 and 24 h incubation post-treatment with B[a]P, 100 µL of Griess reagent (Sigma, St. Louis, USA) was added to each well and incubated in dark for 15 min. The intensity of the color developed was estimated at 540 nm with the help of a Benchmark plus 96-well ELISA plate reader (Biorad, CA, USA). The amount of nitrite accumulated was calculated (in µM) from a standard curve constructed with different concentrations of sodium nitrite.

### Cytokine bead array

The BD Mouse Inflammation cytokine bead array (CBA) kits were used to quantitatively measure cytokine levels in BV-2 cell culture supernatants and mouse whole-brain lysates. 50 µL of bead mix containing a population of beads with distinct fluorescence intensities that have been coated with capture antibodies for cytokines, and 50 µL of culture supernatant or whole-brain lysate were incubated together, along with equal volume of PE-conjugated detection antibodies, for 2 h at room temperature, in dark. The beads were then washed and resuspended in 300 µL of supplied 1X wash buffer. The beads were acquired using Cell Quest Pro Software in FACS Calibur and analyzed using BD CBA software (Becton Dickinson, San Diego, CA). Standard curve was prepared by incubating 50 µL of mouse inflammation standards with 50 µL of bead mix and PE-conjugated detection antibodies [35].

### Immunoblotting

BV-2 cells were plated in 60-mm plates at a density of 10^5^ cells/mL (after triplicate counts with a haemocytometer) and were treated as described above. Cells from each treatment groups were washed twice with ice-cold 1X PBS, and then lysed with lysis buffer. Protein levels were determined by Bradford method. Twenty micrograms of each sample was electrophoresed on polyacrylamide gel and transferred onto a nitrocellulose membrane. After blocking with 7% skimmed milk, the blots were incubated overnight at 4°C with primary antibodies against iNOS (Chemicon, USA), SOD-1 (Santa Cruz Biotechnology, CA, USA), TRX (AB Frontiers, Korea; a kind gift from Dr. Ellora Sen, NBRC) and phosphoP38 MAP kinase (Cell Signalling, USA) at 1∶1000 dilutions. After extensive washes in PBS–Tween, blots were incubated with appropriate secondary antibodies conjugated with peroxidase (Vector Laboratories, CA, USA). The blots were again washed in PBS–Tween and processed for development using chemiluminescence reagent (Millipore, USA). The images were captured and analyzed using Chemigenius, Bioimaging System (Syngene, Cambridge, UK) [38]. The blots were stripped and reprobed with anti-β-tubulin (Santa Cruz Biotechnology, USA) to determine equivalent loading of samples.

### Immunofluorescent study of cells and tissues

For immunofluorescent staining of primary neurons, the cells were plated in poly-D-lysine coated Labtek chamber slide (Labtek, Roskilde, Denmark) were fixed in 4% paraformaldehyde for 20 min after B[a]P treatment, and then blocked with 5% serum. The slides were then incubated overnight in humid chamber at 4°C with primary antibodies against beta III tubulin (1∶250; Promega Corporation, Madison, WI, USA) and glial fibrillary acidic protein (GFAP; 1∶500; Dako, Glostrup, Denmark). After washing with 1X PBS, the slides were incubated with appropriate secondary antibodies. After final washings the chambers were removed and slides mounted with DAPI. The slides were observed under Zeiss Axioplan 2 Fluorescence microscope (Zeiss, Gottingen, Germany).

To show activation of primary microglia following treatment with B[a]P, cells were seeded onto Labtek chamber slides and treated with 0.2 µM of B[a]P for 48 h. The cells were then processed as described above. After blocking the cells were incubated overnight in humid chamber at 4°C with primary antibodies against CD11b (1∶250; Chemicon, USA), a microglial marker. On the next day the slides were washed and incubated with appropriate secondary antibody. After final washes, the chambers were removed and the slides were mounted with DAPI and observed under Zeiss Axioplan 2 Fluorescence microscope.

For immunofluorescent staining of intracellular iNOS and phosphoP38 MAP kinase, primary microglia were cultured and then seeded onto Labtek chamber slides. Following treatment with 0.2 µM B[a]P treatment for 3 and 12 h, cells were processed for staining as described above. The cells were incubated overnight, with primary antibodies against CD11b and iNOS (1∶250; Chemicon, USA) or against CD11b and phosphoP38MAP kinase (1∶250; Cell Signaling, USA), in humid chambers, at 4°C. Following incubation with appropriate secondary antibodies and final washes, the chambers were removed, the slides were mounted with DAPI and observed under Zeiss Axioplan 2 Fluorescence microscope.

BALB/c mice were intraperitoneally (i.p) injected with B[a]P at doses of 100 µg, 1 mg, and 10 mg per kg body weight, for 4 days. Control animals were injected with DMSO. Animals from control and B[a]P treated groups were sacrificed after 4 days of treatment, after anesthetizing them with ketamine hydrochloride, administered i.p at a dose of 40 mg/kg body weight. After repeated transcardial perfusion with ice cold 1X PBS, the brains were excised and processed for cryostat sectioning. To label activated microglia and activated astrocytes, 20 micron thick sections were incubated overnight at 4°C with rabbit anti-Iba-1 (Wako, Osaka, Japan) and rabbit anti-glial fibrillary acidic protein (GFAP) (Dako, Glostrup, Denmark), respectively at dilutions of 1∶500. After washes, slides were incubated with appropriate secondary antibodies conjugated with fluorescein (Vector Laboratories, CA, USA) and following final washes, sections were mounted with DAPI. The slides were observed under Zeiss Apotome microscope and Zeiss Axioplan 2 fluorescence microscope (Zeiss, Gottingen, Germany), respectively.

### Statistical analysis

Statistical analysis was performed using SIGMASTAT software (SPSS Inc., Chicago, IL, USA). Data were compared between groups using one-way analysis of variance followed by Fisher's post hoc test. Differences upto p<0.05 were considered significant.

## Results

### B[a]P does not cause cytotoxicity to primary neurons or neuronal cell lines, directly

**Figure 1:**
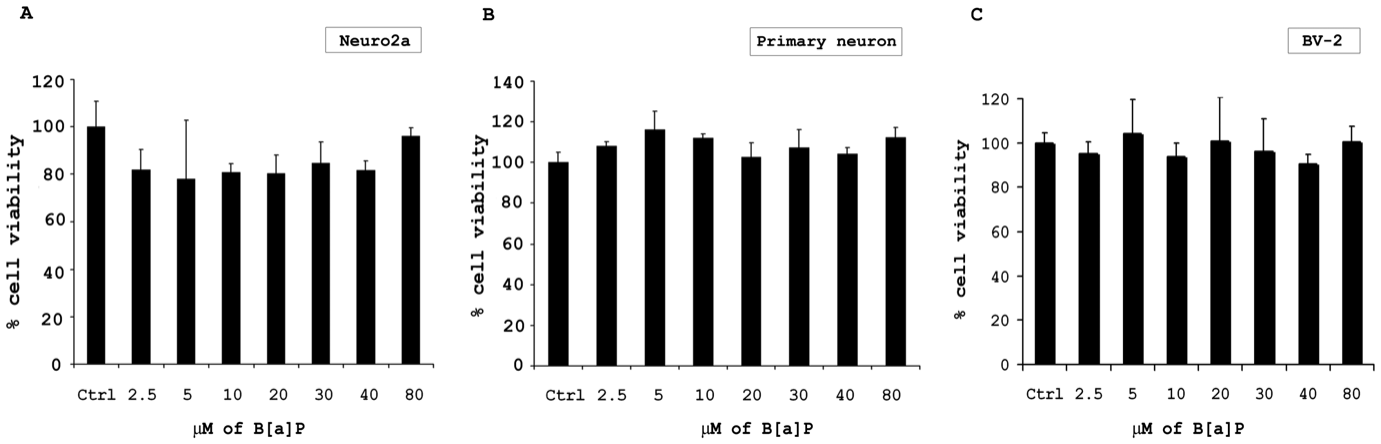
B[a]P treatment does not cause cytotoxicity in murine neuroblastoma cell line, primary cortical neurons or microglial cells. Mouse neuroblastoma Neuro2a (N2a) cells were plated onto 96-well plates, in triplicates, and treated with varying doses of B[a]P. After 48 h incubation, MTS assay was performed that showed that there was no significant amount of cell death in any B[a]P treated groups (A). Similar experiment, when repeated on murine primary cortical neuron, grown on poly-D-lysine coated 96-well plate in triplicates, showed similar results (B). Data represents Mean ± SD of three independent experiments. Murine microglial cell (BV-2), cultured in 96-well plates in triplicates, were treated with varying doses of B[a]P for 48 h. MTS assay was then performed to evaluate cell viability. Results show that there was no significant difference in viability of B[a]P treated microglial cells when compared to control cells (C).

Earlier reports have indicated that B[a]P can be transported and metabolized in the brain [26], [27], apart from other tissues in the body. However, whether B[a]P causes direct neuronal death has not been clearly described. The dose response study using varying doses of B[a]P (as low as 2.5 µM to as high as 80 µM) on murine neuroblastoma cell line (N2a) and murine primary cortical neurons, seeded in 96-well culture plates, revealed that the cell viability in all treatment groups after 48 h of B[a]P administration was 100±20%. The changes in viability, as shown by the MTS assay, were not found to be significantly different from the control cells (*Fig. 1A & B*). Light microscopic images as well of immunofluorescent staining of control and 80 µM B[a]P treated primary neurons did not show any marked difference (*Fig. S1A & B*).

### Treatment with B[a]P leads to activation of murine microglial cells but no cytotoxicity

Microglia are the brains' defense against external pathogens as well as help in the internal clearance of debris. Like neurons or neuronal cell lines, microglia seems to be immune to cytotoxic damage following B[a]P treatment. MTS assay performed on B[a]P treated microglial cell line showed clearly that there was no significant cell death *(* *Fig. 1C* *)*. We found that following treatment with B[a]P there was microglial activation. Light microscopic imaging of BV-2 showed that even after 48 h of incubation, B[a]P treated cells showed morphological signs of activation (*Fig. S1C*). Immunofluorescent staining of B[a]P treated primary microglia for CD11b, a microglial marker, also showed that the cells indeed showed an activated morphology (*Fig. S1D*).

### B[a]P causes elevation of ROS production in microglial cells and modulates intracellular stress protein levels

**Figure 2:**
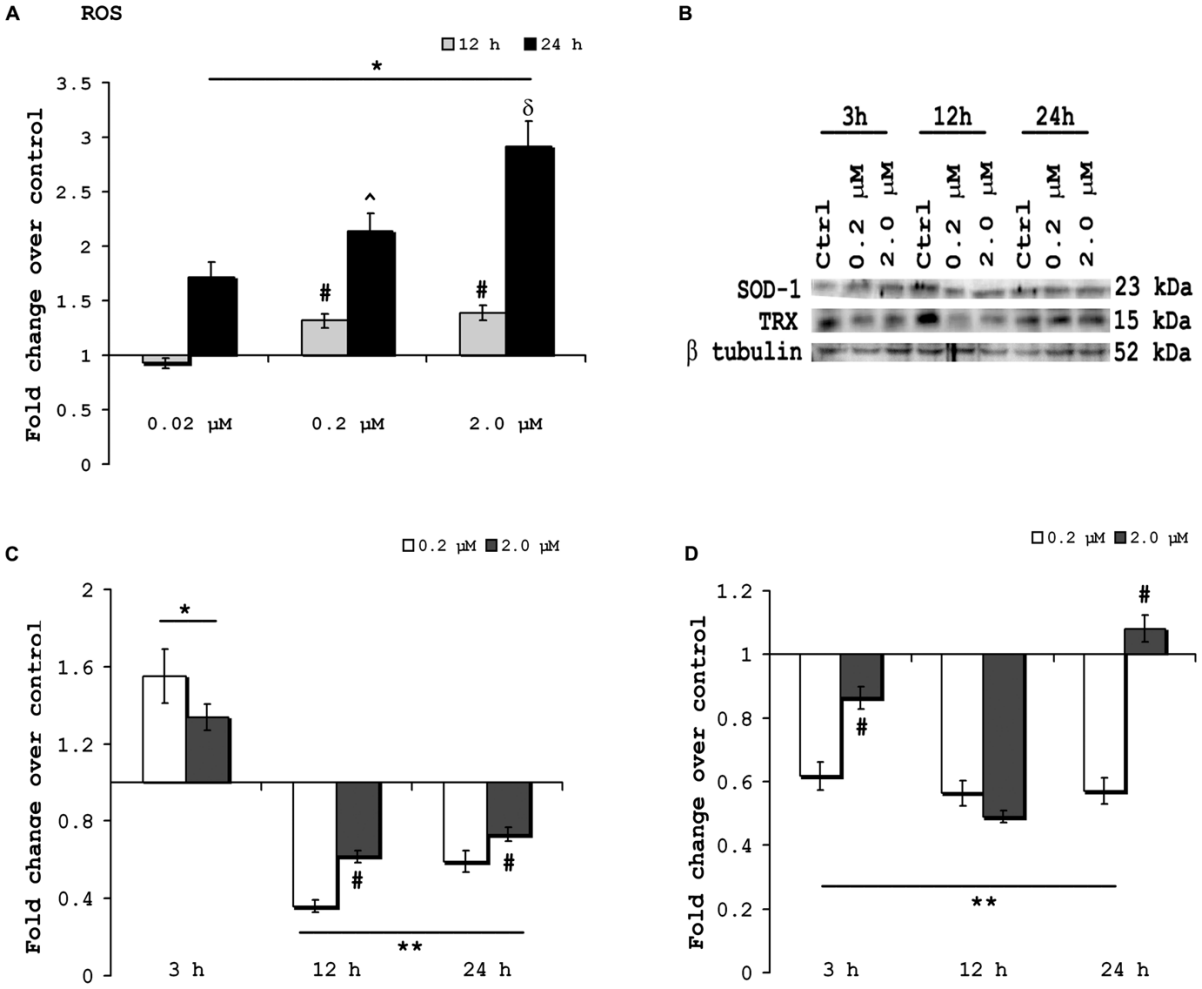
Intracellular ROS is increased after B[a]P treatment and that also affects stress protein levels. BV-2 cells were treated with 0.02 µM, 0.2 µM and 2.0 µM of B[a]P and incubated for 12 h and 24 h. Following incubation, cells were collected and treated with 5 µM solution of CM-H2DCFDA followed by incubation in dark at room temperature for 45 min. Cells were then lysed and centrifuged at 13000 rpm for 20 min at 4°C. Supernatant obtained was used for fluorimetric estimation of ROS using a Varioskan Flash multimode reader. Results show that intracellular ROS levels were significantly elevated in all B[a]P treated groups at either time points except in the 0.2 µM group, after 12 h treatment. The ROS level was significantly higher in 0.2 µM and 2.0 µM groups as compared to 0.02 µM group, 12 h post treatment. After 24 h of treatment, it was observed that ROS levels in 2.0 µM group was significantly higher than that in 0.2 µM group; moreover ROS level in 0.2 µM group was higher than 0.02 µM group. Data is represented as Mean ± SD of fold changes over control from triplicate sets of experiments. ^*^ p<0.01 for all treatment groups when compared to respective time matched controls; ^#^ p<0.01 for 0.2 µM and 2.0 µM groups as compared to 0.02 µM group, 12 h post treatment; ^∧^ p<0.01 for 0.2 µM group as compared to 0.02 µM group in 24 h post treatment sample; ^δ^ p<0.01 for 2.0 µM group as compared to 0.2 µM group in 24 h post treatment sample (A). Levels of intracellular stress proteins- SOD-1 and TRX- following treatment with varying doses of B[a]P were determined by western blotting (B). Results show that after 3 h of B[a]P treatment SOD-1 levels were significantly higher in both 0.2 µM and 2.0 µM groups as when compared to the control. However, SOD-1 levels were significantly decreased in 12 h and 24 h post treatment samples, irrespective of the dose. It was also observed that ROS levels were comparatively higher in 2.0 µM groups than in 0.2 µM groups in both 12 h and 24 h samples (C). TRX levels were found to be significantly decreased in all B[a]P treated groups except in 2.0 µM group, 24 h post infection. TRX levels were also, significantly higher in 2.0 µM group as compared to 0.2 µM group in 3 h and 24 h post infection samples (D). Data is represented as Mean ± SD of fold changes over control from triplicate sets of experiments. * p<0.01 for 0.2 µM and 2.0 µM B[a]P treated groups when compared to control; ** p<0.01 for 0.2 µM and 2.0 µM B[a]P treated groups when compared to control; ^#^p<0.01 for 2.0 µM group as compared to 0.2 µM group.

Microglial activation results in elevation of intracellular ROS levels in them [40]. To see whether B[a]P treatment affects intracellular ROS levels, BV-2 cells were treated with 0.02 µM, 0.2 µM, 2.0 µM B[a]P for 12 h and 24 h. ROS was estimated fluorimetrically by addition of the cell permeable, non-polar, H_2_O_2_-sensitive probe CM-H2DCFDA. It was observed that 12 h post treatment with B[a]P, ROS levels were significantly increased in 0.2 µM and 2.0 µM treated groups (p<0.01); after 24 h treatment significant increase was observed in all three treatment groups when compared to control (p<0.01). In 24 h treated samples the ROS levels in 0.2 µM group was significantly higher than 0.02 µM treated sample, and that in 2.0 µM group was significantly higher than 0.2 µM group (p<0.01) (*Fig. 2A*).

Intracellular level of superoxide dismutase 1 (SOD-1) was found to be significantly elevated after 3 h treatment with 0.2 µM and 2.0 µM B[a]P, when compared to time matched, control cells (p<0.01). SOD-1 was found to be slightly elevated in the 0.2 µM B[a]P treated sample than the 2.0 µM B[a]P sample, though this was not statistically significant. However in 12 h and 24 h post treatment sample, SOD-1 levels were found to be significantly decreased than control in all B[a]P treated groups (p<0.01). Also, it was observed that in 12 h and 24 h post treatment samples, SOD-1 level was significantly greater in 2.0 µM than in 0.2 µM group (p<0.01) (*Fig. 2B & C*). Thioredoxin (TRX) levels were found to be significantly decreased than control in all B[a]P treated groups at all time points except 2.0 µM group 24 h post infection (p<0.01). Also TRX levels were significantly higher in 2.0 µM group as compared to 0.2 µM group in 3 h and 24 h post infection samples (p<0.01) (*Fig. 2B & D*).

### B[a]P causes increased release of nitric oxide from microglial cells and modulates intracellular inducible nitric oxide synthase levels

**Figure 3:**
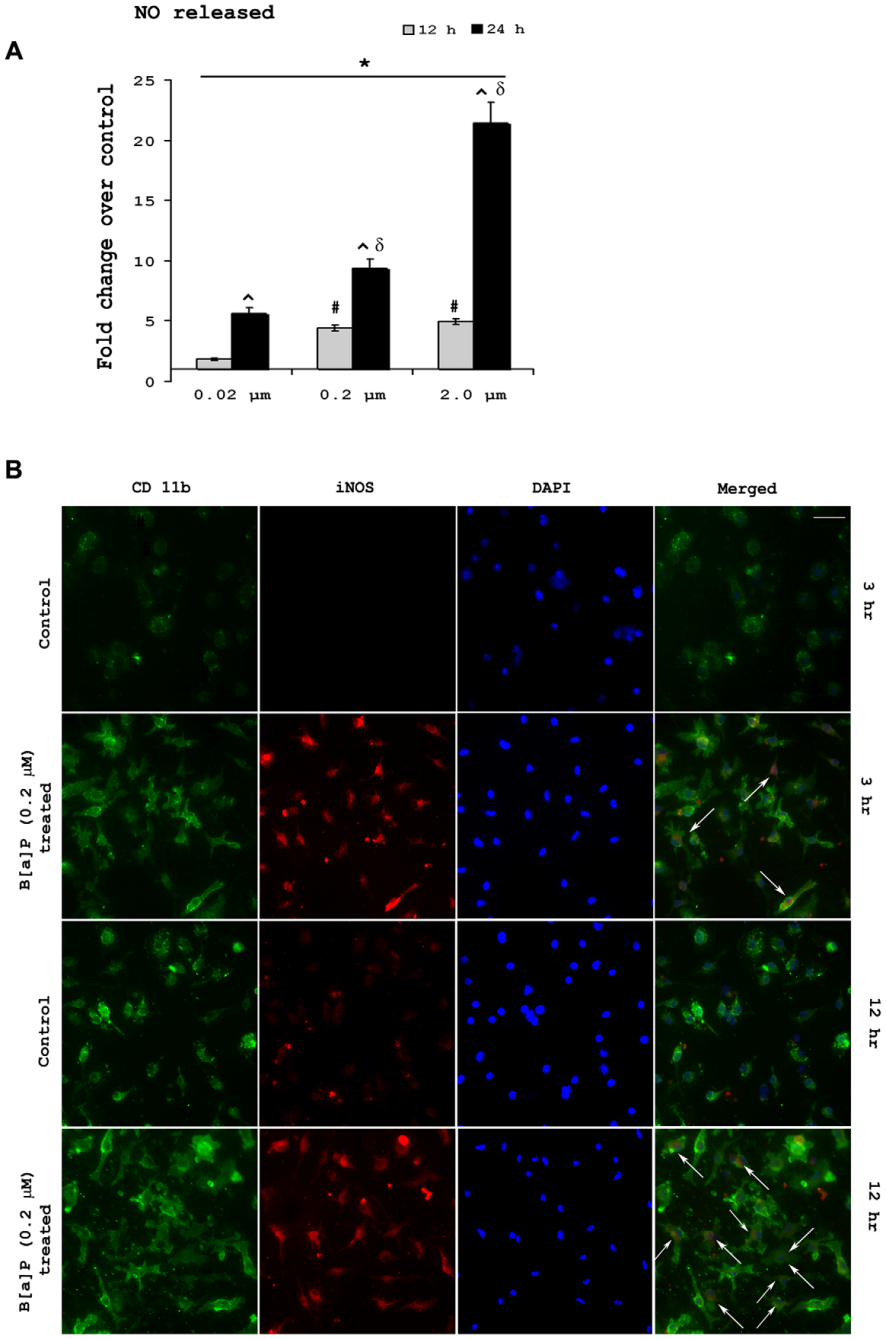
B[a]P treatment causes elevated NO release from microglia and also modulates intracellular iNOS levels. BV-2 cells were were treated with B[a]P at doses of 0.02 µM, 0.2 µM and 2.0 µM for 12 h and 24 h. NO released by the cells were then estimated using Greiss reagent from the culture supernatants. Results show that NO released from B[a]P treated microglia was significantly higher than control microglia that were treated with DMSO only. The NO released from 0.2 µM and 2.0 µM groups were significantly higher than 0.02 µM groups after both 12 h and 24 h of treatment. Fold increase of NO release from B[a]P treated microglia over control was greater after 24 h as compared to 12 h. Results are Mean ± SD of fold changes over control from triplicate sets of experiments. * p<0.01 for all treatment groups when compared to respective controls; ^#^ p<0.01 for 0.2 µM and 2.0 µM B[a]P treated group when compared to 0.02 µM B[a]P group, 3 h post treatment; ^∧^ p<0.01 for fold increases after 24 h when compared to fold increases after 12 h treatment in all groups; ^δ^ p<0.01 for 0.2 µM B[a]P treated group when compared to 0.02 µM B[a]P treated group; ^ε^ p<0.01 for 2.0 µM B[a]P treated group when compared to 0.2 µM B[a]P treated group (A).To assess whether iNOS expression was altered within microglial cells following B[a]P treatment, primary microglia cultured in chamber slides were treated with 0.2 µM B[a]P for 3 h and 12 h. Time matched control cells received DMSO. The cells were costained for CD11b, a microglial marker and iNOS and images were captured in a Zeiss Axioplan 2 fluorescence microscope. The images show that there is increased expression of iNOS following B[a]P treatment. This increase was more prominent after 12 h as compared to 3 h treatment (B). White arrow denotes cells in which activation is visibly prominent. Images are representative of triplicate sets. Magnification is 20×; scale bar corresponds to 50 µ.

Activated microglia are known to produce nitric oxide (NO) that is capable of causing neuronal damage [41]. We found that upon B[a]P treatment, there was significantly increased NO release from BV-2 cells after 12 or 24 h of treatment when compared to untreated time matched controls (p<0.01). Moreover, it was observed that fold increase of NO released over control after 24 h treatment with B[a]P, was significantly higher, when compared to 12 h treatment groups (p<0.01). In 24 h post treatment group, it was observed that the increase in NO release seemed to be dose dependent. NO released from 0.2 µM group was significantly higher (∼1.7 fold) than 0.02 µM group, whereas that from 2.0 µM group was significantly higher (∼2.3 fold) than 0.2 µM group (p<0.01) (*Fig. 3A*).

Immunocytochemical study performed to observe intracellular inducible nitric oxide synthase (iNOS) levels following B[a]P treatment were achieved by staining primary microglia with anti-iNOS antibody. Primary microglia were cultured in chamber slides and treated with 0.2 µM B[a]P for 3 h and 12 h. Time matched controls were treated with DMSO. Co-staining with anti-iNOS and anti-CD11b antibody showed that after 3 h of treatment, there was expression of iNOS within the microglia. After 12 h treatment, the expression was visibly higher than that observed in 3 h post treatment cells (*Fig. 3B*).

### Intracellular phosphoP38 MAP kinase levels are modulated as a result of B[a]P treatment

**Figure 4:**
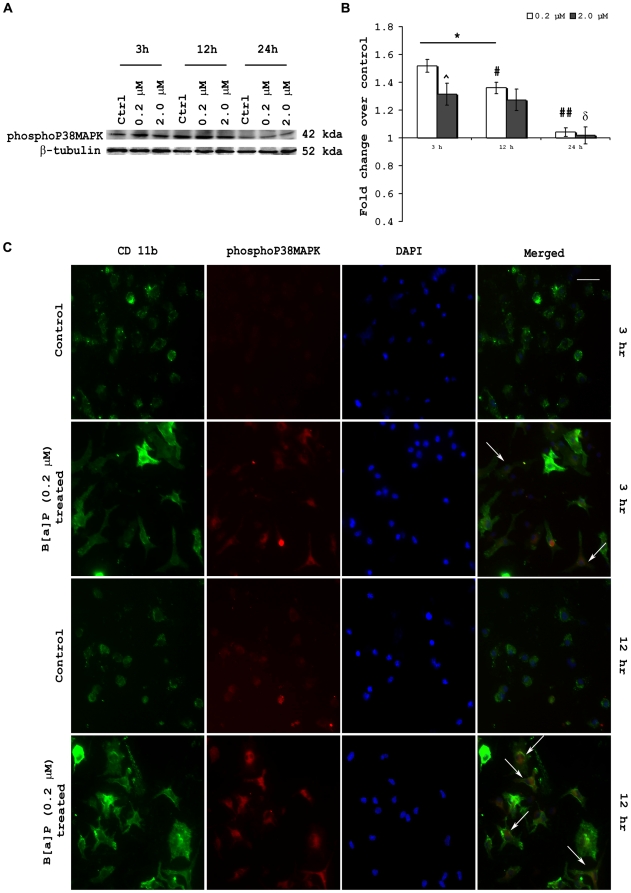
B[a]P treatment results in modulation of intracellular phosphop38 MAP kinase. Western blotting was performed with protein harvested from BV-2 cells after 3 h, 12 h and 24 h treatment with 0.2 µM and 2.0 µM B[a]P (A). PhosphoP38 MAP kinase levels were significantly elevated in 3 h post treatment groups and also in 0.2 µM treated group after 12 h. After 24 h, there was no significant difference in the phosphoP38 MAP kinase levels in either B[a]P treated group when compared to time matched control. However, it was observed that phosphoP38 MAP kinase level in 0.2 µM B[a]P treated group after 12 h, was significantly decreased as compared to that in 3 h post treatment group. Similarly, phosphoP38 MAP kinase level in 0.2 µM B[a]P treated group after 24 h, was significantly decreased as compared to that in 12 h post treatment group. PhosphoP38 MAP kinase level was also significantly reduced in 24 h post treatment 2.0 µM group when compared to 3 h and 12 h post treatment groups of the same concentration (B). Data is represented as Mean ± SD of fold changes over control from triplicate sets of experiments. * p<0.01 for all groups as compared to control; # p<0.05 for 12 h 0.2 µM group as compared to 3 h 0.2 µM group; ## p<0.01 for 24 h 0.2 µM group as compared to 12 h 0.2 µM group; δ p<0.01 for 24 h 2.0 µM group as compared to 12 h 2.0 µM group. Primary microglia were stained with anti-phosphoP38 MAP kinase and anti-CD 11b antibodies, after 3 h and 12 h B[a]P treatment. The results show that upon treatment with B[a]P there was an increased intracellular expression of phosphoP38 MAP kinase. This was also observed after 12 h treatment. Moreover, in the 12 h treated cells, the phosphoP38 MAP kinase's fluorescent signals seemed to be localized towards the nucleus (C). White arrow denotes cells in which activation is visibly prominent. Images are representative of triplicate sets. Magnification is 20×; scale bar corresponds to 50 µ.

Protein harvested from BV-2 cells after 3 h, 12 h and 24 h treatment with 0.2 µM and 2.0 µM B[a]P were processed for immunoblotting to detect intracellular level of phosphoP38 MAP kinase. The results show that phosphoP38 MAP kinase levels were significantly elevated in 3 h post treatment groups and also in 0.2 µM treated group after 12 h (p<0.01). After 24 h, there was no significant difference in the phosphoP38 MAP kinase levels in either B[a]P treated group when compared to time matched control. However, it was observed that phosphoP38 MAP kinase level in 0.2 µM B[a]P treated group after 12 h, was significantly decreased as compared to that in 3 h post treatment group (p<0.05). Similarly, phosphoP38 MAP kinase level in 0.2 µM B[a]P treated group after 24 h, was significantly decreased as compared to that in 12 h post treatment group (p<0.01). PhosphoP38 MAP kinase level was also significantly reduced in 24 h post treatment 2.0 µM group when compared to 3 h and 12 h post treatment groups of the same concentration (p<0.01) (*Fig. 4A & B*).

Immunofluorescent staining to detect intracellular phosphoP38 MAP kinase after B[a]P treatment was also carried out on primary microglia. Cells cultured on chamber slides were treated with 0.2 µM B[a]P for 3 h and 12 h following which they were fixed with 4% paraformaldehyde, and stained with anti-phosphoP38 MAP kinase antibody. The results show that upon treatment with B[a]P there was an increased intracellular expression of phosphoP38 MAP kinase. This was also prominently observed after 12 h treatment. (*Fig. 4C*).

### B[a]P treated microglia secrete proinflammatory cytokines

**Figure 5:**
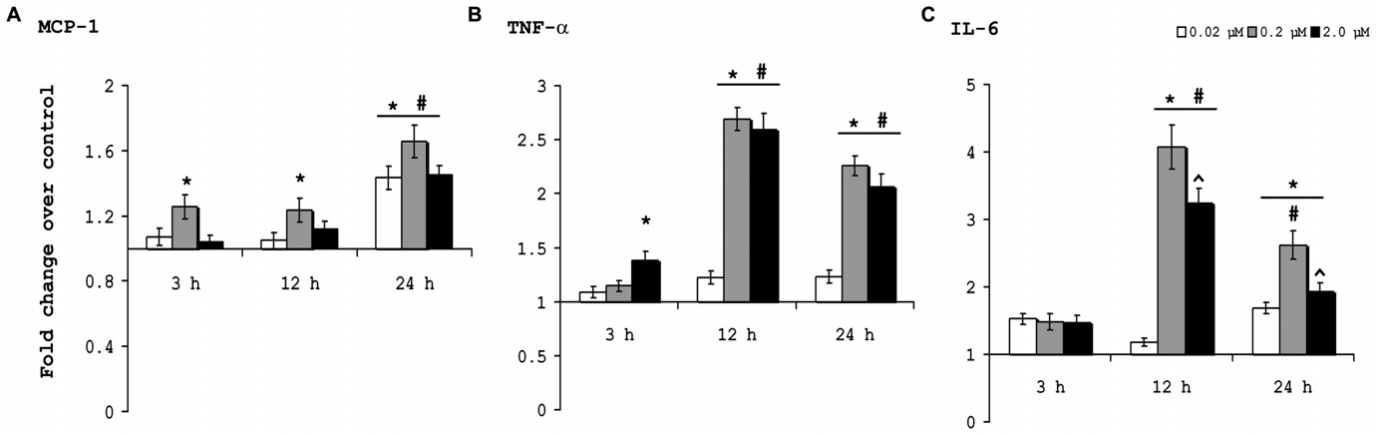
Proinflammatory cytokine levels are increased in culture supernatants following B[a]P treatment. Quantitative estimation of proinflammatory cytokines released in the culture media by B[a]P-treated BV-2 cells were carried out with the help of BD CBA kits. Data was acquired and analyzed in BD FACS calibur instrument with the help of Cell Quest Pro software. MCP-1 was was found to be significantly increased than control, only after treatment with 0.2 µM B[a]P in 3 h and 12 h post treatment samples. In 24 h samples, all groups showed significantly increased MCP-1 level than control (A). TNF-α was found to be increased significantly in 2.0 µM B[a]P treated sample after 3 h and in 0.2 µM and 2.0 µM B[a]P treated samples after 12 h and 24 h when compared to control and 0.02 µM treated sample (B). IL-6 levels were not found to be significantly different in any B[a]P treated group, after 3 h of treatment. After 12 h, the 0.2 µM and 2.0 µM groups showed significant increase in IL-6 when compared to control and also when compared to 0.02 µM treated. IL-6 level in 2.0 µM group was found to be significantly lesser than that observed in 0.2 µM group. After 24 h, IL-6 levels were found to be significantly elevated in all B[a]P treated groups. However, the IL-6 level in 2.0 µM group was found to be significantly lesser than that observed in 0.2 µM group (p<0.01) (C). Data is represented as Mean ± SD of fold changes over control from triplicate sets of experiments. * p<0.01 for significant increases over control; # P<0.01 for significant increases over 0.02 µM groups; ^∧^ p<0.01 for 2.0 µM as compared to 0.2 µM treated group.

BV-2 were cultured in 60 mm plates and were treated with varying doses of B[a]P for 3 h, 12 h, and 24 h. CBA was performed using kits with the culture supernatants to quantitate the amount of cytokines released into the supernatant from the cells. The MCP-1 level was found to be significantly increased than control, only after treatment with 0.2 µM B[a]P for 3 h (p<0.01). Similar result was also observed after 12 h treatment, but after 24 h, MCP-1 levels were found to increase significantly in all B[a]P treated groups when compared to their time matched control (p<0.01). However, in this case also, the 0.2 µM treated group showed maximal release of MCP-1 (*Fig. 5A*). TNF-α levels showed no significant difference in 0.02 µM and 0.2 µM B[a]P treated groups after 3 h of treatment, but in the 2.0 µM treated group, there was a significant increase (p<0.01). After 12 h of treatment, the 0.2 µM and 2.0 µM groups showed significant increase in TNF-α when compared to control and also when compared to 0.02 µM treated group (p<0.01). Similar results were obtained after 24 h treatment (*Fig. 5B*). IL-6 levels were not found to be significantly different in any B[a]P treated group, after 3 h of treatment. After 12 h, the 0.2 µM and 2.0 µM B[a]P treated groups showed significant increase in IL-6 secretion when compared to control cells, and also when compared to 0.02 µM treated group (p<0.01). Also, the IL-6 level in 2.0 µM group was found to be significantly lesser than that observed in 0.2 µM group (p<0.01). After 24 h, IL-6 levels were found to be significantly elevated in all B[a]P treated groups (p<0.01). However, the IL-6 level in 2.0 µM group was found to be significantly lesser than that observed in 0.2 µM group (p<0.01) (*Fig. 5C*).

### Activation of microglia by B[a]P treatment leads to bystander neuronal death

**Figure 6:**
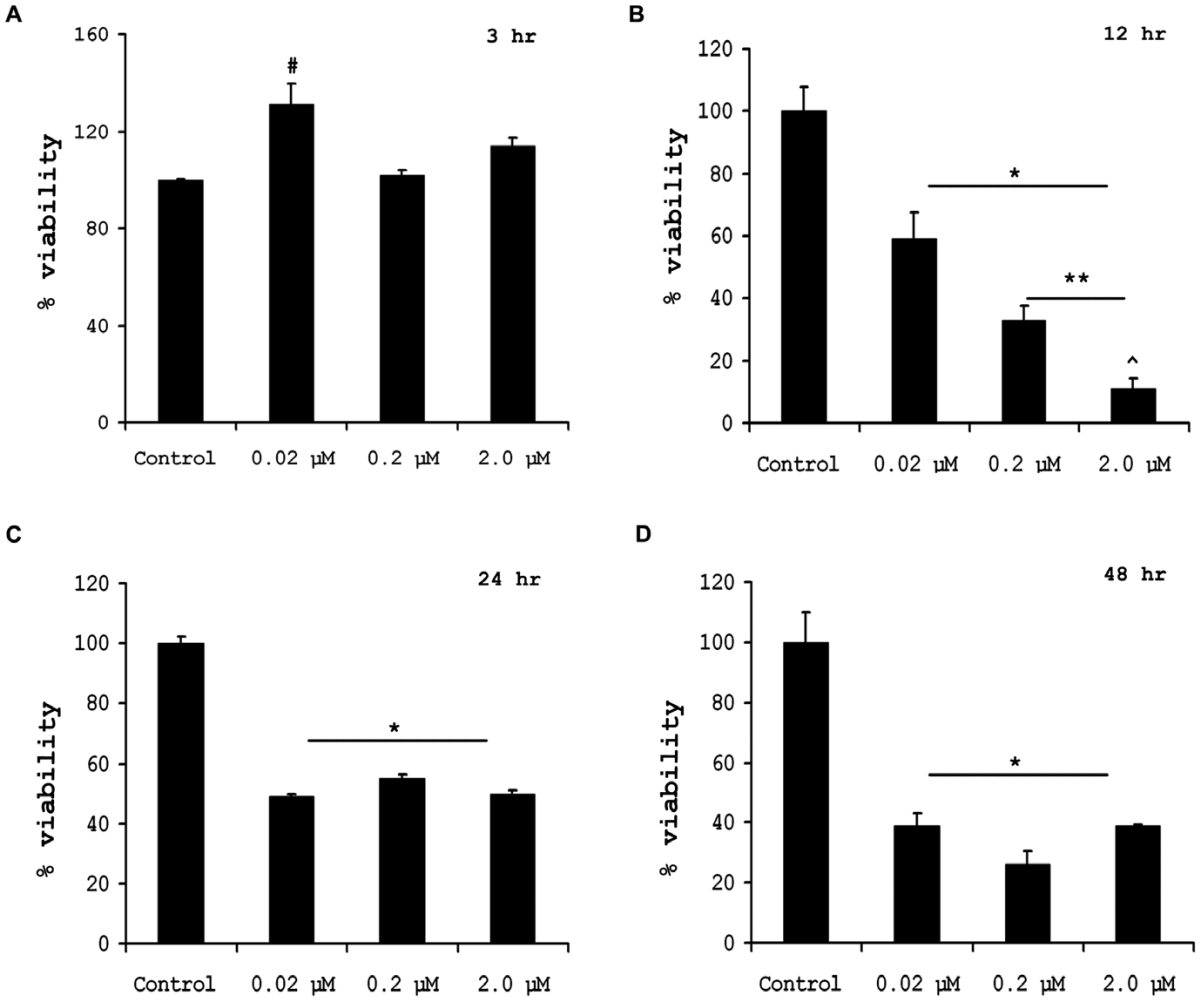
Bystander neuronal death caused by B[a]P treated microglia. To assess whether B[a]P treated microglia causes bystander neuronal death *in vitro*, BV-2 cells were treated with 0.02 µM, 0.2 µM and 2.0 µM of B[a]P for 3, 12, 24 and 48 h. After each time point the culture supernatant were collected form each treatment group, filtered and stored at −20°C. Time matched controls were simultaneously maintained that were treated with DMSO. Primary cortical neurons were then treated with these culture supernatants and incubated for 48 h, followed by MTS assay. Results show that on addition of 3 h post treatment supernatants, there was no significant difference in cell viability in 0.2 µM and 2.0 µM groups, when compared to control. However, the cells in 0.02 µM treatment group showed an increased viability (A). On addition of 12 h post treatment supernatants, there was significant reduction of cell viability in all treatment groups as compared to the group that was treated only with control. Moreover, it was observed that the viability of 0.2 µM group was significantly lesser than that in 0.02 µM group. Similarly the viability in 2.0 µM group was significantly lesser than in 0.2 µM group (B). After addition of 24 h post treatment supernatant on primary neurons, the viability was found to be significantly decreased in all treatment groups when compared to control, but there was no significant difference between the viability of the treatment groups (C). Addition of 48 h post treatment supernatants on primary neurons also resulted in significant decrease of cell viability as compared to control, but there was no significant difference between the B[a]P treated groups (D).Data represented as Mean ± SD of three independent experiments; ^#^ p<0.01 for 0.02 µM treatment group when compared to control; ^*^ p<0.01 for all treatment groups compared to control; ** p<0.01 for 0.2 µM treatment group when compared to 0.02 µM treatment group; ^∧^ p<0.01 for 2.0 µM treatment group when compared to 0.2 µM treatment group.

Activated microglia is known to produce an array of proinflammatory mediators that are in turn deleterious for surrounding neurons in the CNS [9], [42], [43]. To check whether B[a]P causes bystander neuronal death by activating microglia, we treated murine microglial cell BV-2 with varying doses of B[a]P, viz. 0.02 µM, 0.2 µM and 2.0 µM, for 3 h, 12 h, 24 h and 48 h. The control comprised of culture supernatants from time matched DMSO-treated BV-2 cells. The culture supernatants were collected and stored at −20°C after each time period. After collection of the 48 h sample, filtered aliquots of these supernatants were added on mouse primary cortical neurons that had been cultured in triplicates, in poly-D-lysine coated 96-well plates followed by incubation for 48 h. MTS assay performed to determine cell viability post treatment with B[a]P treated BV-2 supernatants. Results show that in the cells on which 3 h culture supernatant was added, there was no significant cell death in 0.2 µM and 2.0 µM treatment groups but the 0.02 µM treated groups showed increased viability (p<0.05) (*Fig. 6A*). Primary cortical neurons on which 12 h post treatment culture supernatants were added showed significantly reduced viability in all groups, when compared to control (p<0.01). The viability of primary neurons on which 0.2 µM B[a]P treated BV-2 culture supernatant was added showed a significantly reduced viability when compared to 0.02 µM treated group (p<0.01); similarly 2.0 µM group showed lesser viability than 0.2 µM treated group (p<0.01) (*Fig. 6B*). In primary neurons on which 24 h post treatment supernatants were added, all groups showed significantly reduced viability than the control (p<0.01), however there were no differences between 0.02 µM, 0.2 µM and 2.0 µM treated groups (*Fig. 6C*). Similar observations were recorded in primary neurons on which 48 post treatment supernatants were added (*Fig. 6D*).

### B[a]P causes upregulation of proinflammatory cytokines in brain *in vivo*

**Figure 7:**
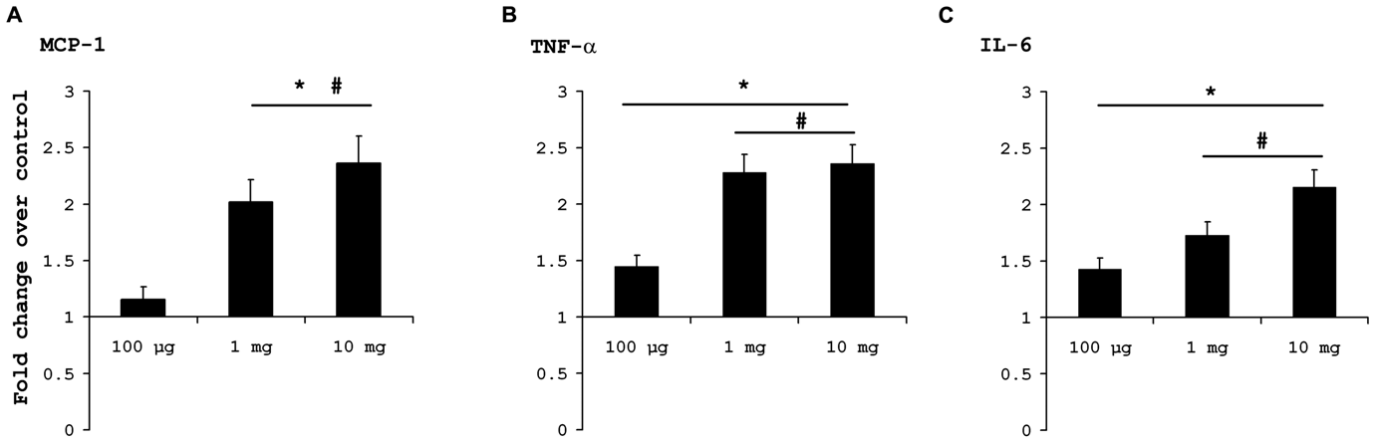
Treatment with B[a]P causes elevation of proinflammatory cytokines in the brain. Mice were administered 100 µg, 1 mg and 10 mg of B[a]P, intraperitoneally for 4 consecutive days and then sacrificed. The brain was excised out and homogenized. The homogenates were used for determination of proinflammatory cytokine content using CBA kit. Results show that MCP-1 was increased in 1 mg and 10 mg treated groups as compared to control. Also, the MCP-1 level in 10 mg group was higher than that in 1 mg group (A). TNF-α levels were significantly increased after B[a]P administration in all groups, as compared to control. TNF-α level in 1 mg and 10 mg groups were also significantly higher than 100 µg group (B). IL-6 levels showed a significant increase in all B[a]P treated groups compared to control. IL-6 level in 1 mg and 10 mg group was significantly higher than that in 100 µg group; also it was significantly higher in 10 mg group when compared to 1 mg group (C). Data is represented as Mean ± SD of fold changes over control from six animals of each group. * p<0.01 for changes over control; ^#^ p<0.01 for changes over 100 µg group; ^∧^ for changes over 1 mg group.

To find out whether B[a]P also causes inflammatory changes *in vivo*, we administered B[a]P at doses of 100 µg, 1 mg and 10 mg per kg body weight (henceforth referred as 100 µg, 1 mg and 10 mg treated groups), intraperitoneally, to BALB/c mice for 4 consecutive days. Proinflammatory cytokine levels in brain tissue homogenates were estimated using CBA kits. Results show that after 4 days of treatment there was an increased MCP-1 level in 1 mg and 10 mg B[a]P treated groups (p<0.01) when compared to either control or 100 µg treated groups. Moreover, MCP-1 was significantly higher in 10 mg group, when compared to 1 mg (p<0.01) (*Fig. 7A*). TNF-α levels were significantly increased in all B[a]P treated groups when compared to control (p<0.01). Also, TNF-α level in 1 mg and 10 mg groups was significantly higher than 100 µg group (p<0.001) (*Fig. 7B*). IL-6 level was found to be elevated in all B[a]P treated groups when compared against control (p<0.01). The level in 1 mg and 10 mg group was significantly higher than that in 100 µg group (p<0.01); also it was significantly higher in 10 mg group when compared to 1 mg group (p<0.01) (*Fig. 7C*).

### B[a]P causes microglial activation and astrogliosis in the brain

**Figure 8:**
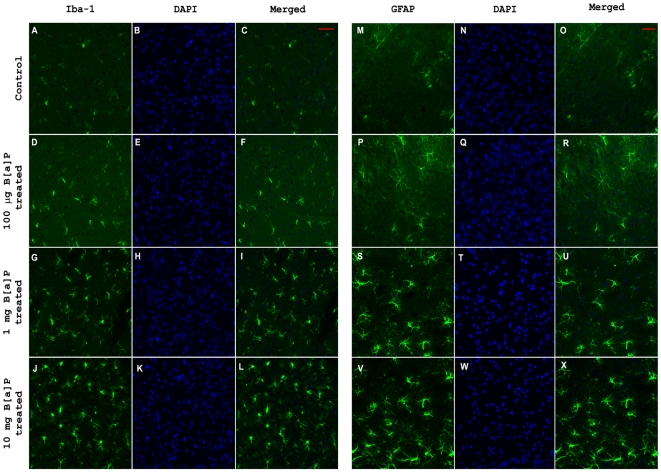
Activation of microglia and astrogliosis in vivo due to B[a]P treatment. Microglial activation following B[a]P treatment was observed by staining the cryosections for Iba-1. The figure clearly show that B[a]P treatment successfully caused microglial activation (D–L), as compared to control (A–C), though it was more prominent in 1 mg and 10 mg treated animals (G–I & J–L). Astrogliosis was also observed after 4 day treatment with B[a]P, if compared with control (M–O). In the 1 mg (S–U) and 10 mg (V–X) treated groups the morphology of the activated cells were more robust than compared to 100 µg treated group (P–R). The figures represent cryosections of a mouse brain of each group as a representative of six animals from each group. Images of sections stained for Iba-1 were acquired using a Zeiss Apotome microscope, while those that were stained for GFAP were acquired using a Zeiss Axioplan 2 fluorescence microscope. Magnification is 20×; scale bars correspond to 50 µ.

Microglia and astrocytes have both been reported to be involved with inflammatory reactions taking place within the brain [44], [45]. To check whether B[a]P treatment causes activation of microglia or astrocytes, 20 µ thick cryostat sections of brain from control and B[a]P treated animals were analyzed immunohistochemically for Iba-1, a marker for microglia and GFAP, a marker for astrocytes. Images show that after 4 day treatment there was microglial activation in all B[a]P treated groups (*Fig. 8D–L*). Robust changes in morphology of the microglia were observed in 1 mg and 10 mg treated groups (*Fig. 8G–L*). Astrogliosis was also observed in all B[a]P treated groups (*Fig. 8P–X*); however in 1 mg and 10 mg groups, the change in morphology was prominent (*Fig. 8S–X*). The number of astrocytes was also found to be highest in 10 mg treated group, if compared with all other treatment groups. The figures represent cryosections of a mouse brain from each group as a representative of six animals from each group.

## Discussion

From an ecological aspect, B[a]P as a pollutant has been under investigation for quite some time [13], [46], [47]. It is relevant in environmental and occupational settings where specific populations are chronically exposed to B[a]P that is released from hazardous waste sites, or via food, water or tobacco, or during direct occupational exposures. B[a]P has been shown to be metabolized and accumulated in hepatic cells [48] and has also been found in the brain at least after 24 h post administration [27]. Investigations in the past has reported neuronal death following B[a]P administration [28], [29], [30], [31], though the actual mechanism by which the death occurs is unknown. In this study, using neuroblastoma cell lines and primary cortical neurons, it was found that even after using B[a]P at doses as high as 80 µM, there was no significant decrease in cell viability. Microscopic imaging also failed to demonstrate any evidence of morphological changes of the cells. Taken together, these results clearly show that doses of B[a]P up to 80 µM are not directly toxic to neurons. As the cells had been incubated with B[a]P for upto 48 h, it can be safely assumed that whatever metabolite of B[a]P had been formed within the cells, was also not cytotoxic to neurons. Thus, ruling out the direct damage to neurons due to toxicity, the other significant way by which neuronal death can occur is due to the generation of an inflammatory milieu within the brain. Microglia are resident macrophages of the CNS whose overexpression or dysregulation is instrumental in generating inflammatory response within the brain. We suspected that changes in microglia due to administration of B[a]P may be a contributing factor that is responsible neurotoxicity. To test this hypothesis, we performed survivality assay after addition of B[a]P to murine microglial cell line BV-2, in same doses as applied to neuroblastoma cells or primary neurons, and observed no significant decreases in cell survivality. When observed under microscope, the microglia seemed to be activated (data not shown). The doses of B[a]P used for survivality assays, had been chosen at random, to test a wide range. However, for the study to achieve physiological significance, such doses had to be used that would seem relevant to the real life scenario. The lowest detectable concentration of B[a]P common to human experience, for statistically significant mutations in human cells, has been found to be 0.02 µM [49]. We chose this value as our lowest dose and two other subsequent doses that are 10-folds and 100-folds higher, (0.2 µM and 2.0 µM respectively), for the *in vitro* experiments. On application of these doses, it was found that there were clear morphological signs of activation in BV-2. On treating primary microglia with 0.2 µM B[a]P for 48 h, the cells were found to assume an ameboid morphology that is also indicative of its activated state.

The intracellular ROS levels are an important characteristic in the cellular responses to external stress. Increase in ROS levels initiates various responses within the cell, including damage to proteins, DNA and lipid [50]. When intracellular ROS was measured from the microglia, after treatment with varying doses of B[a]P for different time periods, we found that there was significant increase. Correspondingly, SOD-1 and TRX levels were found to be decreased within the microglia. SOD-1 has been reported to ameliorate the oxidative stress generated by wide array of causes and also protects the cells from stress-induced apoptosis. However, we see that B[a]P induced oxidative stress is not being countered by SOD-1. TRX is also another important antioxidant component within the cell that is necessary to ameliorate elevated ROS. TRX scavenges ROS by itself and in cooperation with peroxiredoxin and also plays a crucial role in the redox regulation of transcription factors for the expression of various genes. TRX levels were also found to be reduced after B[a]P treatment. Thus it seems that the intracellular antioxidant machinery of the cell is not capable of countering the oxidative stress generated due to B[a]P.

NO produced from the B[a]P treated microglia was found to be increased substantially than untreated cells. NO, a major signaling molecule has been shown to induce oxidative stress and trigger apoptosis in neuronal cells [51]. The magnitude of NO release was found to be greatly increased after 12 h and 24 h post treatment with B[a]P. NO released from these microglia can thus act on neurons to initiate killing. We also found that intracellular iNOS expression was also increased correspondingly. Primary microglia, when stained for iNOS, showed increased expression of iNOS after 12 h which suggests that B[a]P treatment causes its upregulation. Thus, these data clearly shows that following B[a]P treatment, iNOS is induced within microglia that results in increased production of NO from the cells.

The p38 MAPK pathway has been shown to be activated by ROS [52]. Studies have also shown that once p38 MAPK pathway is activated, it is capable of damaging antioxidative system that further increases intracellular ROS levels. P38 MAPK pathway is also known to be involved in the production of various cytokines, in response to stress. Thus, intracellular levels of phosphoP38 MAPK were found to be elevated within the microglia following treatment with B[a]P for 3 h, 12 h and 24 h. Interestingly, it was also observed that the phosphoP38 MAPK, though higher than untreated cells, tends to decrease as time progresses. This may be explained by the fact that nuclear translocation of phosphoP38 MAPK occurs to initiate transcription for the synthesis of proinflammatory cytokines, though further studies are required to substantiate this aspect.

The levels of proinflammatoy cytokines such as MCP-1, TNF-α and IL-6 were estimated from microglial culture supernatants after treatment with B[a]P for different time periods. A general increase in the secretion of the cytokines from 12 h onwards was observed that persisted even after 24 h, but no significant difference was found after 48 h (data not shown). After 3 h treatment, only 0.2 µM and 2.0 µM treated groups showed some difference in case of MCP-1 and TNF- α, respectively. This is plausible as the cellular machinery needs time to produce these cytokines and subsequently release them. Also, taking into consideration the time factor, after 48 h we failed to find any significant difference in the cytokines levels. Interestingly, we also found that treatment with the 0.2 µM dose showed maximal cytokine (MCP-1 and IL-6) production from the cells. The 0.2 µM doses may be ideal for optimal response from the microglia, but it would not be prudent to come to such conclusions without further studies and thus, this phenomenon, currently remains enigmatic. IL-6 is known to induce neuronal death by excessive activation of NMDA receptors [53]. TNF-α induces inflammatory reactions and can induce death signaling via receptors present on neurons [54] or may do so by abnormal AMPA receptor trafficking [55]. MCP-1 does not directly affect neuronal death, however it has been shown to be associated with microglial activation [56] leading to bystander neuronal death. Thus, the inflammatory milieu created by the secretion of these proinflammatory cytokines could be detrimental to neurons.

On the basis of above observations, we were interested to see whether culture supernatants of B[a]P treated microglia, could actually affect neuronal survivality. On addition of the supernatants onto primary neurons, it was seen that there was significant reduction of neuronal viability. This effect was prominent from the 12^th^ hour post treatment samples. This shows that following treatment with B[a]P, the neurotoxic factors that are actually responsible for killing the neurons, have accumulated in sufficient amount after 12 h of treatment. This effect was observed till the addition of 48 h post treatment supernatant, indicating that the factors that are responsible for the death of the neurons, are being secreted till that time. Thus the combination of ROS, NO and proinflammatory cytokines seems to act in a synergistic way to cause bystander death to neurons.

*In vivo* experiments showed that after 4 days of B[a]P treatment, proinflammatory cytokine levels in brain were found to be significantly elevated. MCP-1, IL-6 and TNF-α levels were found to be increased in a dose dependent manner, indicating microglial activation in the brain following B[a]P administration. To confirm this assumption, brain sections were stained for activated microglia which were found to be present prominently in 1 mg and 10 mg B[a]P treated animals and also to a lesser extent in 100 µg treated group. Similarly, astrogliosis was also observed in all three B[a]P treated groups.

In conclusion it may be said that B[a]P induces microglial activation by increasing intracellular ROS levels, down regulating antioxidant proteins and activating p38MAP kinase pathway. This leads to release of proinflammatory cytokines and NO which in turn initiates an inflammatory cascade in the CNS, resulting in bystander neuronal death *(Fig. S2)*. In this study, we have shown for the first time the role of B[a]P in inducing neuroinflammation in the CNS and its possible outcome. Increasing environmental pollution levels have led us to believe that presence of PAHs should also increase simultaneously. Thus from today's perspective this study is assumes significance as this study involves a potent environmental pollutant, B[a]P.

## Supporting Information

Figure S1 Photomicrographs showing effect of B[a]P treatment on N2a, primary neurons, BV-2 and primary microglia. Light microscopic images of primary cortical neurons, grown in poly-D-lysine coated chamber slides that were treated with 80 µM of B[a]P, did not reveal any significant morphological alterations when compared to control (A). To confirm this finding, immunofluorescent staining of the B[a]P treated primary neurons were done. The slides were stained for beta III tubulin, a primary neuronal marker, and glial acidic fibrilary protein (GFAP), a marker for activated astrocytes, followed by mounting with DAPI. The image shows that no significant change can be visualized in the B[a]P treated neurons when compared to control (B). The scale bars correspond to 50 µ and magnification is 20×. Light microscopic images of BV-2 after treatment with varying doses of B[a]P for 48 h shows morphological signs of activation at all three doses (C). To see whether primary microglia also became activated due to B[a]P, cells were culture and then seeded onto chamber slides and treated with 0.2 µM B[a]P for 48 h. The slides were then processed to be stained with anti-CD11b antibody and mounted with DAPI. Images were captured using Zeiss Axioplan 2 fluorescence microscope. Figure S1D clearly shows morphological difference between B[a]P treated and untreated cells. Scale bar correspond to 50 µ in both (C) and (D). Magnification is 20× in both figures.
(2.93 MB TIF)

Figure S2 Schematic diagram showing the proposed mechanism of action of B[a]P. B[a]P causes activation of microglia by elevating intracellular ROS levels and subsequently decreases antioxidant protein (SOD-1 & TRX) levels. Expression of iNOS is increased in B[a]P treated microglia that leads to increased production and release of NO from them. The p38MAP kinase pathway is also upregulated by B[a]P. The proinflammatory cytokines and NO released results in generation of an inflammatory milieu that is detrimental for neurons.
(0.09 MB TIF)
