# Supplementary figures and images for: A Common Carcinogen Benzo[a]pyrene Causes Neuronal Death in Mouse via Microglial Activation
Source: PLoS One. 2010 Apr 1;5(4):e9984. doi: 10.1371/journal.pone.0009984 (PMC2848611; doi:10.1371/journal.pone.0009984)

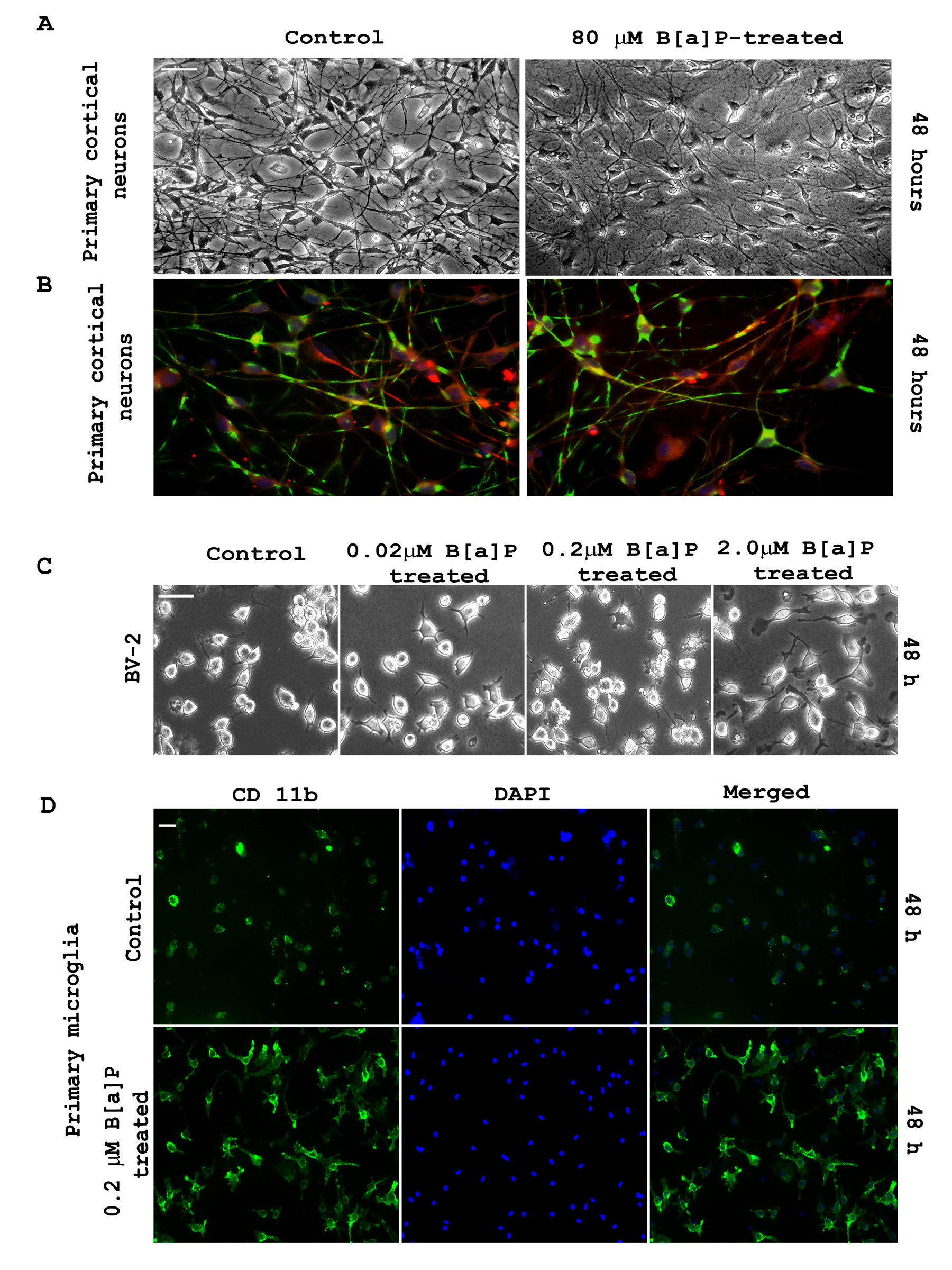

Supplement: Figure S1 — Photomicrographs showing effect of B[a]P treatment on N2a, primary neurons, BV-2 and primary microglia. Light microscopic images of primary cortical neurons, grown in poly-D-lysine coated chamber slides that were treated with 80 µM of B[a]P, did not reveal any significant morphological alterations when compared to control (A). To confirm this finding, immunofluorescent staining of the B[a]P treated primary neurons were done. The slides were stained for beta III tubulin, a primary neuronal marker, and glial acidic fibrilary protein (GFAP), a marker for activated astrocytes, followed by mounting with DAPI. The image shows that no significant change can be visualized in the B[a]P treated neurons when compared to control (B). The scale bars correspond to 50 µ and magnification is 20×. Light microscopic images of BV-2 after treatment with varying doses of B[a]P for 48 h shows morphological signs of activation at all three doses (C). To see whether primary microglia also became activated due to B[a]P, cells were culture and then seeded onto chamber slides and treated with 0.2 µM B[a]P for 48 h. The slides were then processed to be stained with anti-CD11b antibody and mounted with DAPI. Images were captured using Zeiss Axioplan 2 fluorescence microscope. Figure S1D clearly shows morphological difference between B[a]P treated and untreated cells. Scale bar correspond to 50 µ in both (C) and (D). Magnification is 20× in both figures. (2.93 MB TIF) [file pone.0009984.s001.tif]

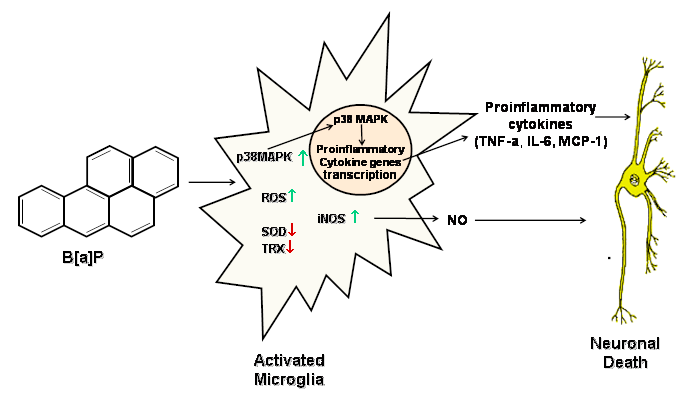

Supplement: Figure S2 — Schematic diagram showing the proposed mechanism of action of B[a]P. B[a]P causes activation of microglia by elevating intracellular ROS levels and subsequently decreases antioxidant protein (SOD-1 & TRX) levels. Expression of iNOS is increased in B[a]P treated microglia that leads to increased production and release of NO from them. The p38MAP kinase pathway is also upregulated by B[a]P. The proinflammatory cytokines and NO released results in generation of an inflammatory milieu that is detrimental for neurons. (0.09 MB TIF) [file pone.0009984.s002.tif]
